# Altered nicotinamide adenine dinucleotide metabolism drives cartilage degeneration and osteoarthritis

**DOI:** 10.1002/ctm2.70513

**Published:** 2025-11-06

**Authors:** Xiaoxin Wu, Xiwei Fan, Manuel Plan, Terra Stark, Tim McCubbin, Roberto A. Barrero, Maria Marinova, Michael J. Bertoldo, Dale M. Goss, Lindsay E. Wu, Ross Crawford, Xinzhan Mao, Indira Prasadam

**Affiliations:** ^1^ Centre for Biomedical Technologies Queensland University of Technology Brisbane Queensland Australia; ^2^ Department of Orthopaedic Surgery The Second Xiangya Hospital Central South University Changsha China; ^3^ Center for AI‐Driven Medical Research Shenzhen Institute of Advanced Technology Chinese Academy of Sciences Shenzhen China; ^4^ Australian Institute for Bioengineering and Nanotechnology (AIBN) The University of Queensland Brisbane Queensland Australia; ^5^ Metabolomics Australia (Queensland Node) Australian Institute for Bioengineering and Nanotechnology (AIBN) The University of Queensland Brisbane Queensland Australia; ^6^ eResearch Office Queensland University of Technology Brisbane Queensland Australia; ^7^ School of Biomedical Sciences University of New South Wales (UNSW) Sydney New South Wales Australia; ^8^ Department of Orthopaedics The Prince Charles Hospital Brisbane Australia

**Keywords:** cartilage, degeneration, NAD^+^, osteoarthritis (OA), PARP14

## Abstract

**Background:**

We previously conducted a comprehensive survey of energy metabolism in osteoarthritis (OA), revealing significant reductions of nicotinamide adenine dinucleotide (NAD^+^) levels in OA cartilage. This study aimed to test whether NAD^+^ deficiency present in OA plays a mechanistic role in disease development.

**Methods:**

We conducted integrative analyses across human, murine, and rat OA models to examine NAD⁺ metabolism and its regulatory enzymes. The impact of pharmacological NAD⁺ augmentation (via nicotinamide mononucleotide (NMN) and nicotinamide riboside (NR)) and genetic overexpression of the NAD⁺ biosynthetic enzyme NMN adenosyltransferase (NMNAT1) was tested in surgical and aging‐related OA models. Expression and function of the NAD⁺‐consuming enzyme poly (ADP‐ribose) polymerase 14 (PARP14) were examined via siRNA knockdown in chondrocytes under inflammatory conditions, coupled with metabolic assays and extracellular matrix gene profiling.

**Results:**

NAD^+^ levels were decreased in human and murine OA, accompanied by upregulation of both the NAD^+^ biosynthetic enzyme Nicotinamide phosphoribosyltransferase (NAMPT) and the NAD^+^ consuming enzyme PARP14. While NAMPT expression was elevated, its effect on total NAD⁺ may be offset by increased NAD⁺ consumption or substrate limitation under inflammatory conditions. Treatment with NAD^+^ precursors and transgenic overexpression of NMNAT1 suppressed cartilage disruption during in aging murine and surgical rat model of OA. Increased expression of PARP14 in OA cartilage contributed to NAD^+^ decline and promoted cartilage degeneration.

**Conclusions:**

This study reveals that dysregulated NAD⁺ metabolism, driven by increased PARP14 consumption, constitutes a potential mechanism underlying OA pathogenesis. Our findings support the concept that enhancing NAD⁺ availability via precursors or biosynthetic pathway modulation may offer disease‐modifying effects at the molecular and histological level. Further investigation is needed to determine the functional and translational implications of targeting this pathway.

**Key points:**

- PARP14 is upregulated in OA cartilage and contributes to NAD⁺ depletion.
- PARP14 silencing restores NAD⁺ levels and represses OA‐related metabolic and matrix‐degrading changes.
- NAD⁺ precursor treatment and NMNAT1 overexpression protect against cartilage degeneration in aging and post‐traumatic OA models.

## Graphical abstract

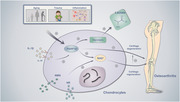

## 1 INTRODUCTION

Osteoarthritis (OA) is the most prevalent degenerative joint disorder, affecting over 500 million people worldwide, and its prevalence continues to rise with aging populations and increasing obesity rates.^1^ OA is a major cause of chronic pain, disability, and reduced quality of life, and it imposes a substantial socioeconomic burden through healthcare costs and loss of productivity.^2^ There are currently no approved disease‐modifying medications for OA. The primary interventions are pain management and referral for arthroplasty in the late stages of OA, which imposes a significant individual and socioeconomic burden. The lack of progress in treating OA is largely due to our incomplete understanding of the mechanisms underlying its initiation and progression. Strategies to overcome OA by reducing inflammation or targeting the removal of senescent cells have been attempted but are yet to be translated into clinical therapeutics for OA. Identifying mechanisms linking OA, senescence, and inflammation could therefore result in potential therapeutic strategies.

Energy metabolism is critically involved in cellular function during chronic inflammation and senescence, both of which are central to OA pathogenesis.^3^ Our previous targeted metabolomics analysis demonstrated that OA chondrocytes exhibit progressive metabolic dysregulation, including a significant decline in nicotinamide adenine dinucleotide (NAD⁺) levels.^4^ NAD⁺ is a key cofactor in energy metabolism and inflammation resolution, and its age‑related decline has been linked to impaired cellular function and tissue degeneration.^5^ In OA, chronic inflammation coincides with increased activity of NAD⁺‑consuming enzymes, such as CD38 and poly(ADP‐ribose) polymerases (PARPs), which can exacerbate NAD⁺ depletion and promote matrix degradation.^6^, ^7^ Chondrocytes are the sole cell type found in cartilage, yet it is unclear whether NAD^+^ metabolism in chondrocytes is altered during the development and pathogenesis of OA. Identifying targetable metabolic pathways, such as NAD⁺ metabolism, therefore offers an opportunity to develop disease‑modifying strategies that could alleviate both the individual and societal impact of OA.

NAD^+^ homoeostasis is a function of both its biosynthesis and its degradation via NAD^+^ consuming enzymes.^8^ Mammalian cells synthesise NAD^+^ in three distinct ways: (1) de novo from tryptophan; (2) via the Preiss‐Handler pathway from nicotinic acid (NA); or (3) via the salvage pathway from nicotinamide mononucleotide (NMN), nicotinamide (NAM) or nicotinamide riboside (NR). For example, NR can be converted to NAD⁺ via an NRK‐independent route, efficiently elevating NAD⁺ levels and providing an alternative mechanism to maintain cellular NAD⁺ homeostasis.^9^ NAD^+^ consuming enzymes such as sirtuins (SIRTs), PARPs, and CD38 counter the rate of NAD^+^ synthesis. The activity of these enzymes is important to various aspects of cellular homoeostasis. For example, sirtuin 1 (SIRT1) can regulate the expression of extracellular matrix (ECM)‐related proteins, as well as act as an anticatabolic, anti‐inflammatory, anti‐oxidative stress, and anti‐apoptotic factor in OA.^10^ In an experimental OA rat model, inhibition of the NAD^+^ consuming enzyme PARP1 dampens the inflammatory response.^7^ Inhibiting CD38 may suppress osteoarthritis development and associated pain after joint injury in mice.^6^ Thus, targeting NAD‐dependent enzymes may help maintain cellular NAD^+^ and energy levels, as well as regulate ECM homoeostasis.

Here, we investigated NAD^+^, its synthesis and consumption in human and animal models of OA. We further tested the impact of exogenously administering NAD^+^ precursors on OA progression in vivo and in vitro *models*. Our data suggest NAD^+^ metabolism plays an important role in OA development, and that treatment with NAD^+^ precursors was associated with reduced cartilage degeneration in preclinical models, suggesting a potential avenue for future investigation.

## 2 RESULTS

### 2.1 Altered NAD⁺ homeostasis and precursor accumulation in human OA and ageing cartilage in mice

**FIGURE 1:**
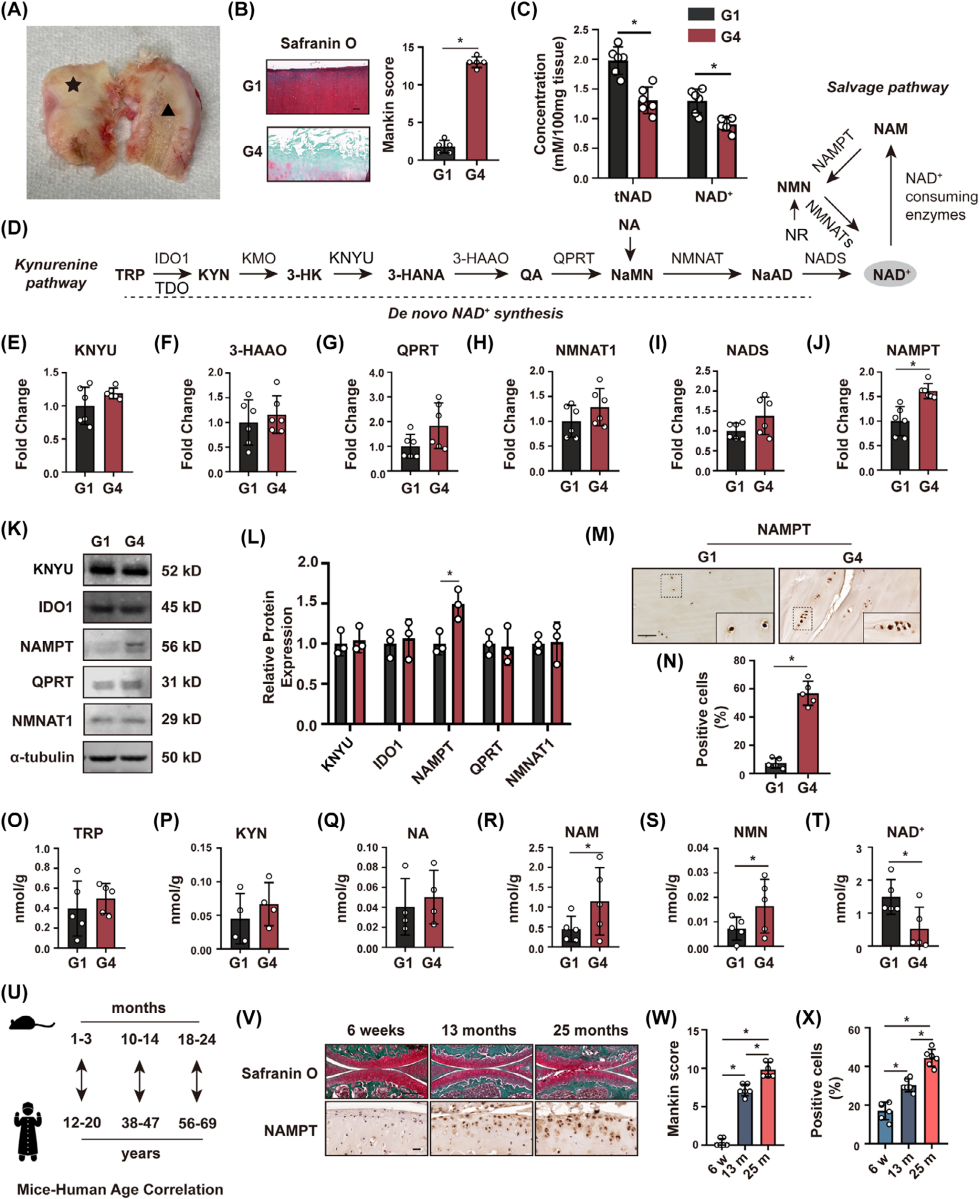
Characterisation of NAD^+^ biosynthesis in human OA and mice ageing cartilage tissue. (A) Classification of human undamaged (G1, ★) and damaged (G4, ▲) cartilage tissue. (B) Modified mankin score and Safranin O/fast green staining for G1 and G4 cartilage. *p < .05; scale bar = 250 µm; *n* = 5. (C) Levels of NAD^+^ and total NAD (tNAD) in G1 and G4 cartilage. *p < .05; *n* = 6. (D) Pathways of Nicotinamide adenine dinucleotide (NAD^+^) biosynthesis diagram. (E–J) NAD^+^ biosynthesis pathway enzyme messenger RNA levels in G1 versus G4 cartilage. *p < .05; *n* = 6. (K) Immunoblotting results of NAD^+^ biosynthesis enzymes in G1 versus G4 cartilage, and (L) quantitative immunoblot analysis. *p < .05; *n* = 3. Nicotinamide phosphoribosyltransferase (NAMPT) immunostaining and quantitative analysis in human G1 and G4 cartilage (M, N). *p < .05; scale bar = 100 µm; *n* = 5. (O–T) LC/MS quantification of metabolites from NAD^+^ biosynthesis pathways in G1 versus G4 cartilage. *p < .05; *n* = 5. (U) Diagram illustrating the age correlation between mice and humans. (V) Safranin O/fast green staining and NAMPT immunostaining in articular cartilage from young, middle‐aged, and elderly mice. Scale bar = 200 µm (top), 20 µm (bottom). (W) Mankin score and (X) NAMPT immunostaining quantitative analysis of articular cartilage from young, middle‐aged, and elderly mice. *n* = 5; *p < .05. All experiments were performed with at least three biological replicates and two technical replicates unless otherwise stated. TRP, tryptophan; IDO1, indoleamine 2, 3‐dioxygenase 1; TDO, tryptophan 2,3‐dioxygenase; KYN, kynurenine; KMO, kynurenine‐3‐monooxygenase; 3‐HK, 3‐hydroxykynurenine; KNYU, kynureninase; 3‐HANA, 3‐ hydroxyanthranilic acid; 3‐HAAO, 3‐hydroxyanthranilic acid 3,4‐dioxygenase; QA, quinolinic acid; QPRT, quinolinate phosphoribosyl transferase; NaMN, nicotinic acid mononucleotide; NA, nicotinic acid; NR, nicotinamide riboside; NMNAT, nicotinamide‐nucleotide adenylyltransferase; NaAD, nicotinic acid adenine dinucleotide; NADS, NAD^+^ synthetase; NMN, nicotinamide mononucleotide; NAM, nicotinamide.

To investigate the role of NAD^+^ in OA, we first determined the level of NAD^+^ biosynthesis in human OA and a mouse ageing model. Following our previous work,^11^ G1 (grade 1) cartilage was isolated from the femur surface with no evidence of cartilage damage, whereas G4 (grade 4) cartilage was derived from the damaged surface with a significantly higher modified Mankin score (Figure 1A and B). We found a significant decrease in NAD⁺ levels and total NAD (tNAD, the sum of both the NAD⁺ and NADH species) in human G4 cartilage when compared to G1 cartilage (Figure 1C), while NADH and the calculated NAD⁺/NADH ratio were non‐significant between G1 and G4 (Figure S1). NAD^+^ is primarily synthesised in mammalian cells via the de novo NAD^+^ synthesis pathway, the Preiss–Handler pathway via NA, and the salvage pathway (Figure 1D). Real‐time quantitative PCR (RT‐qPCR) analysis revealed that NAMPT gene expression was significantly increased in G4 cartilage, whereas other enzymes showed no significant change (Figure 1E–J). The increased expression of NAMPT in G4 cartilage was confirmed by Western blotting and immunohistochemical (IHC) staining (Figure 1K–N). Following that, we measured metabolites from the kynurenine pathway (Figure 1O–Q), with no change observed. Instead, we observed an increase in the NAD^+^ precursors nicotinamide (NAM) and nicotinamide mononucleotide (NMN), along with a decrease in NAD^+^ in G4 cartilage (Figure 1R–T). Together, the accumulation of these NAD^+^ precursors, along with a decline in NAD^+^ levels, suggests declining rates of NAD^+^ biosynthesis at the level of NMN adenosyltransferase (NMNAT) enzymes, which catalyse the conversion of NMN to NAD^+^. To simulate the natural progression of age‐related knee OA in mice, we next assessed cartilage integrity in three age groups of male C57/BL6 mice: young (1–3 months), middle‐aged (10–14 months), and elderly (18–24 months) (Figure 1U).^12^ Histological findings from these mice demonstrated increased NAMPT expression with age, which correlated with the natural age‐related cartilage degeneration in OA progression (Figure 1V–X). Taken together, these findings suggest that despite increased levels of the NAD^+^ biosynthetic enzyme NAMPT, OA development includes a decline in NAD^+^ levels, which is most likely due to declining activity of the subsequent step in NAD biosynthesis, catalysed by NMNAT enzymes.

### 2.2 PARP14 expression was significantly increased in human, mouse, and rat OA cartilage

**FIGURE 2:**
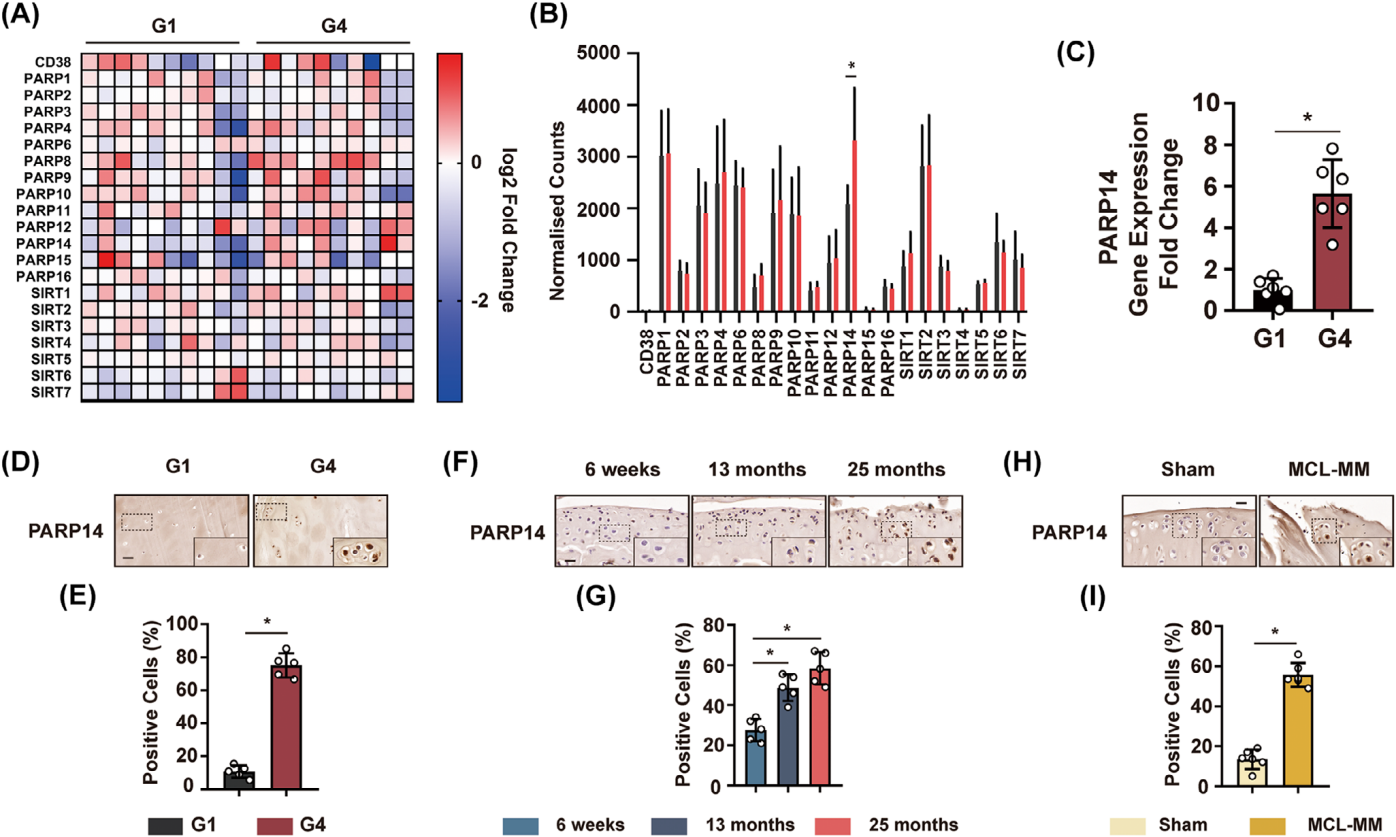
PARP14 is overexpressed in OA cartilage from humans, mice, and rats. (A) Heat map of the gene expression of NAD^+^ consuming enzymes in human G1 and G4 cartilage. *n* = 10. (B) Normalised counts of NAD^+^ consuming enzymes based on gene sequencing. *p < .05; *n* = 10. (C) Real‐time quantitative PCR (RT‐qPCR) analysis of PARP14 gene expression in human G1 versus G4 cartilage. *p < .05; *n* = 6. (D) PARP14 immunostaining on human G1 and G4 cartilage. Scale bar = 50 µm. (E) Quantitative analysis of G1 versus G4 cartilage using PARP14 immunostaining. *p < .05; *n* = 5. (F) PARP14 immunostaining on cartilage from mice aged 6 weeks, 13 months, and 25 months. Scale bar = 20 µm. (G) PARP14 immunostaining quantitative analysis on cartilage from mice aged 6 weeks, 13 months, and 25 months. *p < .05; *n* = 5. (H) Immunostaining for PARP14 on rat cartilage from medial collateral ligament‐medial meniscus (MCL‐MM) OA surgery. Scale bar = 20 µm (bottom). (I) Quantitative analysis of PARP14 immunostaining on rat cartilage following MCL‐MM OA surgery. *p < .05; *n* = 5. All experiments were performed with at least three biological replicates and two technical replicates unless otherwise stated.

Given the decline in NAD^+^ levels during OA, the next key question is whether this is due to an increased rate of NAD^+^ breakdown, and if so, which enzyme(s) were responsible for this. We therefore examined the expression levels of NAD⁺‐consuming enzymes in cartilage, including CD38, PARPs, and Sirtuins, by analysing our previous RNA sequencing data. While PARP1, PARP4, PARP9, PARP14, and SIRT2 were all expressed at a relatively high level in cartilage, PARP14 was the NAD^+^ consuming enzyme among them that was significantly up regulated in G4 cartilage (Figure 2A and B). These RNA‐seq data were confirmed by RT‐qPCR, showing an increase in the level of PARP14 in G4 cartilage (Figure 2C). Immunostaining indicated that PARP14 expression was increased in G4 chondrocytes (Figure 2D and E). We next examined the level of PARP14 in knee cartilage during mouse ageing. As with humans, PARP14 increased significantly in 13‐ and 25‐month mice knee cartilage compared to 6 weeks (Figure 2F and G). Similarly, PARP14 levels were increased in the OA model of rat medial collateral ligament‐medial meniscus (MCL‐MM) surgery (Figure 2H and I). Altogether, these results suggest an increase in the expression of the NAD^+^ consuming enzyme PARP14 in human OA, along with an increase in expression during age‐associated OA in mice, and in a rat OA model.

### 2.3 Pharmacological and transgenic interventions targeting NAD⁺ metabolism attenuate OA development

**FIGURE 3:**
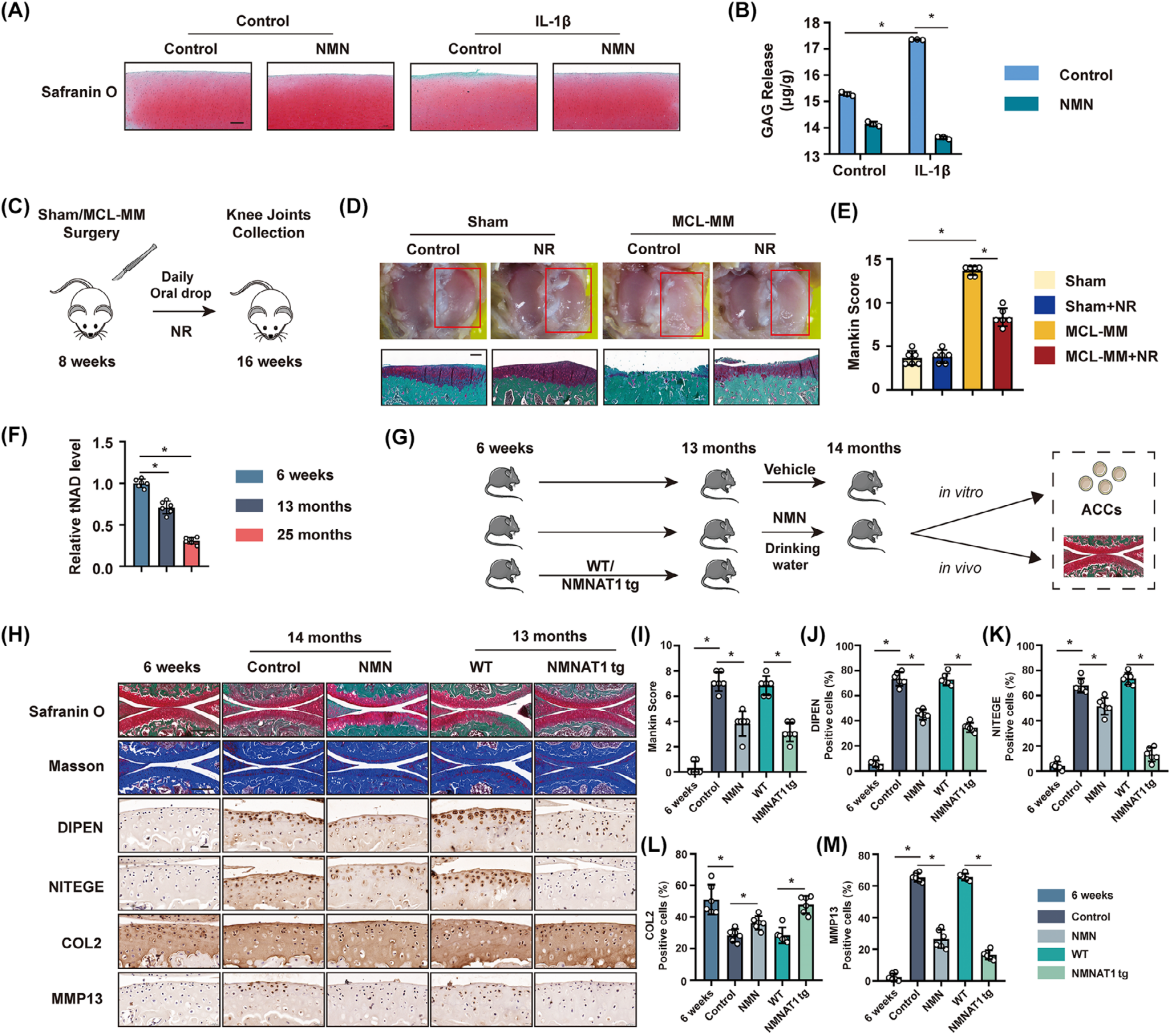
Supplementation with NAD^+^ precursors inhibited the development of OA. (A) Safranin O/Fast Green staining and (B) glycosaminoglycan (GAG) release from normal and IL‐1β‐treated bovine cartilage explants treated with/without 1 mM NMN for 48 h. *n* = 3; *p < .05. Scale bar = 200 µm. (C) Schematic illustrating the protocol for in vivo experiments on rats. Rats were subjected to sham or MCL‐MM surgery for 8 weeks, followed by an 8‐week period with or without 200 mg/kg NR supplementation. *n* = 6. (D, upper) Tibia bone surface images in sham and MCL‐MM rats with and without NR. The red square represents the medial side. (D, bottom) Safranin O/Fast Green on the tibia of sham and MCL‐MM rats with/without NR. Scale bar = 200 µm. (E) Mankin score of the tibia of sham and MCL‐MM rats with/without NR. *p < .05; *n* = 6. (F) tNAD levels in mice knee cartilage after 6 weeks, 13 months, and 25 months. *p < .05; *n* = 6. (G) Schematic depicting the protocol for the in vivo experiment on mice. From 13 months, normal C57/BL6 mice were given 2 g/L NMN in drinking water. We sacrificed 14‐month‐old control, NMN supplemented, 13‐month‐old wild type (WT), and NMNAT1 transgenic (NMNAT1 tg) mice. *n* = 6. (H) Safranin O/Fast Green, Masson, immunostaining, and (I–M) quantitative analysis of knee cartilage from 6‐week‐old, 14‐month‐old, WT, and NMNAT1 tg mice. *p < .05; *n* = 6. Scale bar = 200 µm (upper), 20 µm (bottom). All experiments were performed with at least three biological replicates and two technical replicates unless otherwise stated. ACCs, articular cartilage chondrocytes.

Given the decline in NAD^+^ levels and elevation in the NAD^+^ consuming enzyme PARP14, we next sought to test whether altered NAD^+^ levels played a causative role in mediating OA. To start with, bovine cartilage explants were cultured with/without IL‐1β and NMN for 48 h in the inflammation ex vivo OA model. Our results indicated that IL‐1β treatment significantly increased glycosaminoglycan (GAG) release, whereas NMN supplementation inhibited GAG release (Figure 3A and B). We next used treatment with the NAD^+^ precursors NMN and NR, which have been frequently used to elevate NAD^+^ levels in preclinical and clinical studies.^13^, ^14^ Wistar rats were subjected to sham or MCL‐MM surgery for 8 weeks, followed by daily treatment with NR (200 mg/kg, oral gavage) or vehicle control (Figure 3C). MCL‐MM surgery significantly damaged the medial tibia cartilage, whereas NR supplementation slowed the progression of OA and decreased the Mankin score (Figure 3D and E). Next, we sought to test these findings in the absence of surgical injury, instead examining these changes in the more physiologically relevant context of natural ageing. NAD^+^ levels were significantly reduced in 13‐ and 25‐month‐old mice (Figure 3F). Given this decline in NAD⁺ levels, we established a separate cohort of female C57BL/6 mice to examine the effect of NAD⁺ precursor supplementation. Mice at 13 months of age were randomly assigned to receive either vehicle (regular drinking water) or NMN administered via drinking water at a concentration of 2 g/L for four consecutive weeks (Figure 3G).^15^ NMN treatment led to a dose‐dependent reduction in the severity of cartilage degeneration and decreased the Mankin score compared to controls (Figure 3H and I). In addition to these histological improvements, NMN supplementation in mice reduced the expression of genes associated with OA degeneration while increasing the expression of a chondrogenic protein, Collagen II (COL2) (Figure 3H and J–M). Our in vitro data indicated that NMN had a similar effect on 3D cultured primary chondrocytes from old donors (Figure S1), whereby NMN treatment suppressed the expression of cartilage degeneration genes such as *Mmp1*, *Mmp9*, *Mmp13* (Figure S2D–G), *Dipen* and *Nitege* (Figure S1A and B) while increasing *Col2* chondrogenic gene expression. Our earlier data demonstrated an increase in levels of the NAD^+^ biosynthetic gene NAMPT (Figure 1J–M and V), yet a decrease in NAD^+^. This discrepancy suggests a possible bottleneck at the level of NMNAT enzymes, which catalyse the final step in NAD⁺ biosynthesis. To test this, we next used transgenic animals which over‐express the nuclear localised NMNAT enzyme NMNAT1 (Figure 3G).^15^ NMNAT1 plays a pivotal role in the biosynthesis of NAD⁺, functioning as a key enzyme in the final step of NAD⁺ synthesis.^16^ Overexpression of NMNAT1 has been shown to significantly elevate intracellular NAD⁺ levels, thereby modulating cellular metabolic and redox states.^17^, ^18^ These animals were naturally aged to 13 months, and tissues collected for measurement of age‐associated OA. Notably, NMNAT1‐tg mice were protected against cartilage damage and had a significant reduction in the Mankin score compared to their wild‐type (WT) littermate controls (Figure 3H and I). NMNAT1 overexpression was associated with decreased expression of cartilage degeneration proteins and increased expression of chondrogenic proteins (Figure 3H and J–M). Taken together, these pharmacological and transgenic interventions demonstrate that elevating NAD⁺ levels mitigates cartilage degeneration at the histological and molecular levels in OA models.

### 2.4 PARP14 is required for the metabolism of chondrocytes in an inflammatory environment

**FIGURE 4:**
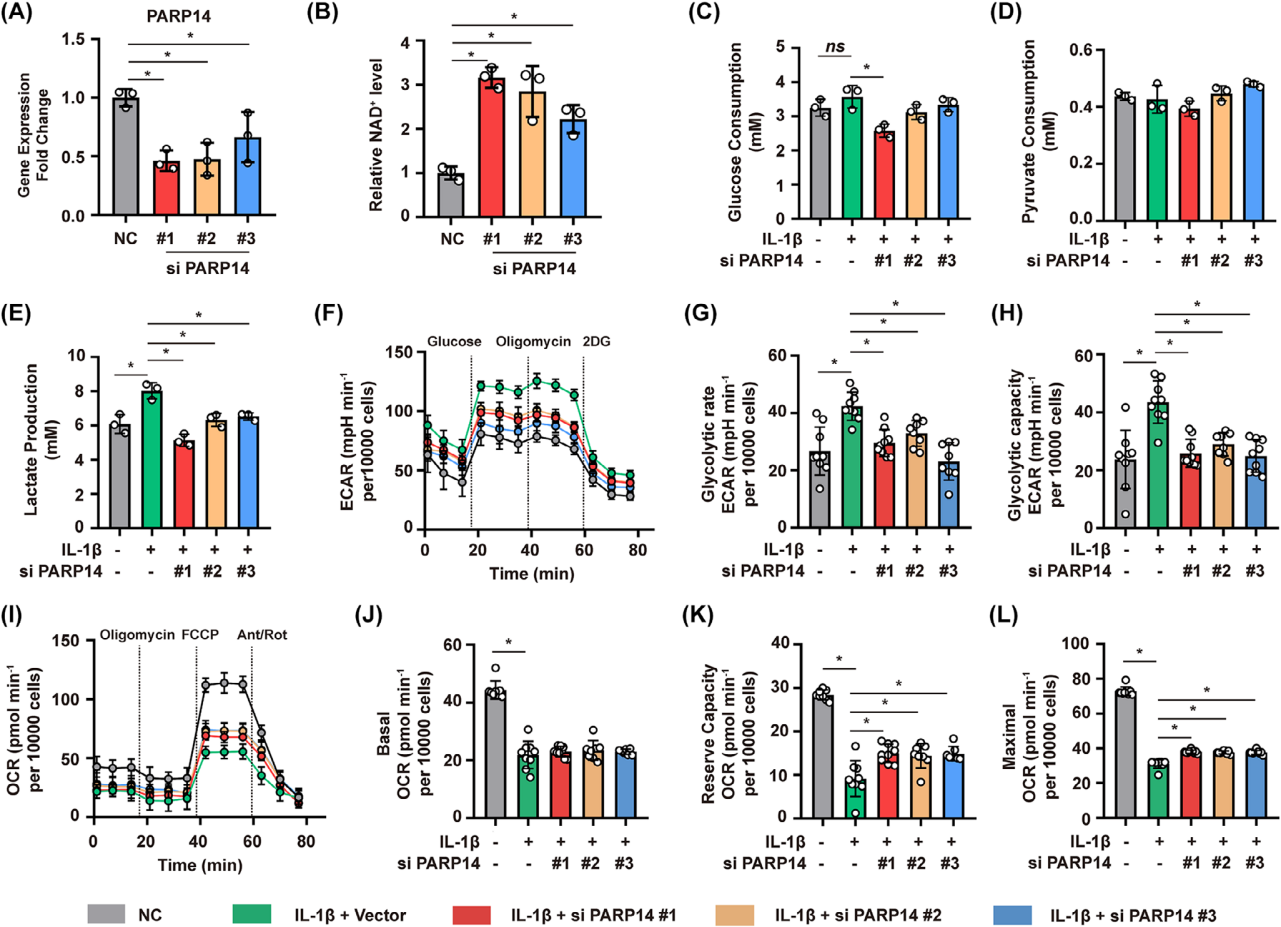
PARP14 is critical for chondrocytes metabolism under an inflammation environment. (A) PARP14 expression in human primary chondrocytes transfected with PARP14 siRNAs or a negative control (NC) siRNA. *n* = 3. (B) NAD^+^ level in chondrocytes 24 h after transfection with PARP14 siRNAs or NC. *n* = 3. (C) Glucose uptake, (D) pyruvate consumption, and (E) lactate production in chondrocytes transfected with PARP14 siRNAs or NC siRNAs. *n* = 3. (F) Seahorse analysis of the extracellular acidification rate (ECAR), glycolysis rate (G), and glycolytic capacity (H) of chondrocytes transfected with PARP14 siRNAs or NC siRNAs. (I) Oxygen consumption rate (OCR), (J) basal OCR, (K) ATP‐linked OCR, and (L) maximal OCR in chondrocytes transfected with PARP14 siRNAs or NC siRNA. *p < .05; *n* = 9. Ns, non‐significant. All experiments were performed with at least three biological replicates and two technical replicates unless otherwise stated.

These results suggested that increased PARP14 activity in OA could lead to a reduction in NAD^+^ levels, which play a causative and reversible role in OA disease progression. To gain a better understanding of the mechanisms underlying PARP14 and chondrocyte function, we examined whether PARP14 affected the energy metabolism of chondrocytes. Three separate PARP14 siRNAs were transfected into primary human chondrocytes (Figure 4A). The effect of PARP14 on chondrocyte viability was then determined using a cell counting kit‐8 (CCK‐8) assay. Interestingly, our results indicated that silencing PARP14 expression had no effect on the viability of chondrocytes after 72 h (Figure S3). In line with the idea that increased PARP14 activity could drive reductions in NAD^+^ levels, PARP14 siRNA silencing significantly increased NAD^+^ levels in chondrocytes after 24 h (Figure 4B). Given the critical role of NAD^+^ as a cofactor for metabolism, we next examined whether PARP14 impacted glycolysis and oxidative phosphorylation. Comparison of untreated controls with IL‐1β–treated cells showed that IL‐1β markedly increased glucose uptake and lactate production, consistent with a glycolytic shift (Figure 4C–E). In chondrocytes exposed to an inflammatory environment, PARP14 silencing resulted in decreased glucose consumption and lactate production compared to the control group, resulting in a higher pyruvate/lactate ratio (Figures 4C–E and S4). Seahorse analysis further confirmed that IL‐1β enhanced glycolytic flux, as indicated by elevated extracellular acidification rate (ECAR), whereas PARP14 silencing suppressed this effect and restored metabolic balance (Figure 4F–H). Mitochondrial stress testing revealed that PARP14 silencing under IL‐1β stimulation did not result in a significant increase in oxygen consumption rate (OCR), but in a substantial increase in reserve capacity and maximal OCR (Figure 4I–L). Together, these findings indicate that PARP14 plays an important role in mediating changes to chondrocyte metabolism including glycolysis during inflammation.

### 2.5 PARP14 regulates the expression of catabolic and anabolic markers in chondrocytes

**FIGURE 5:**
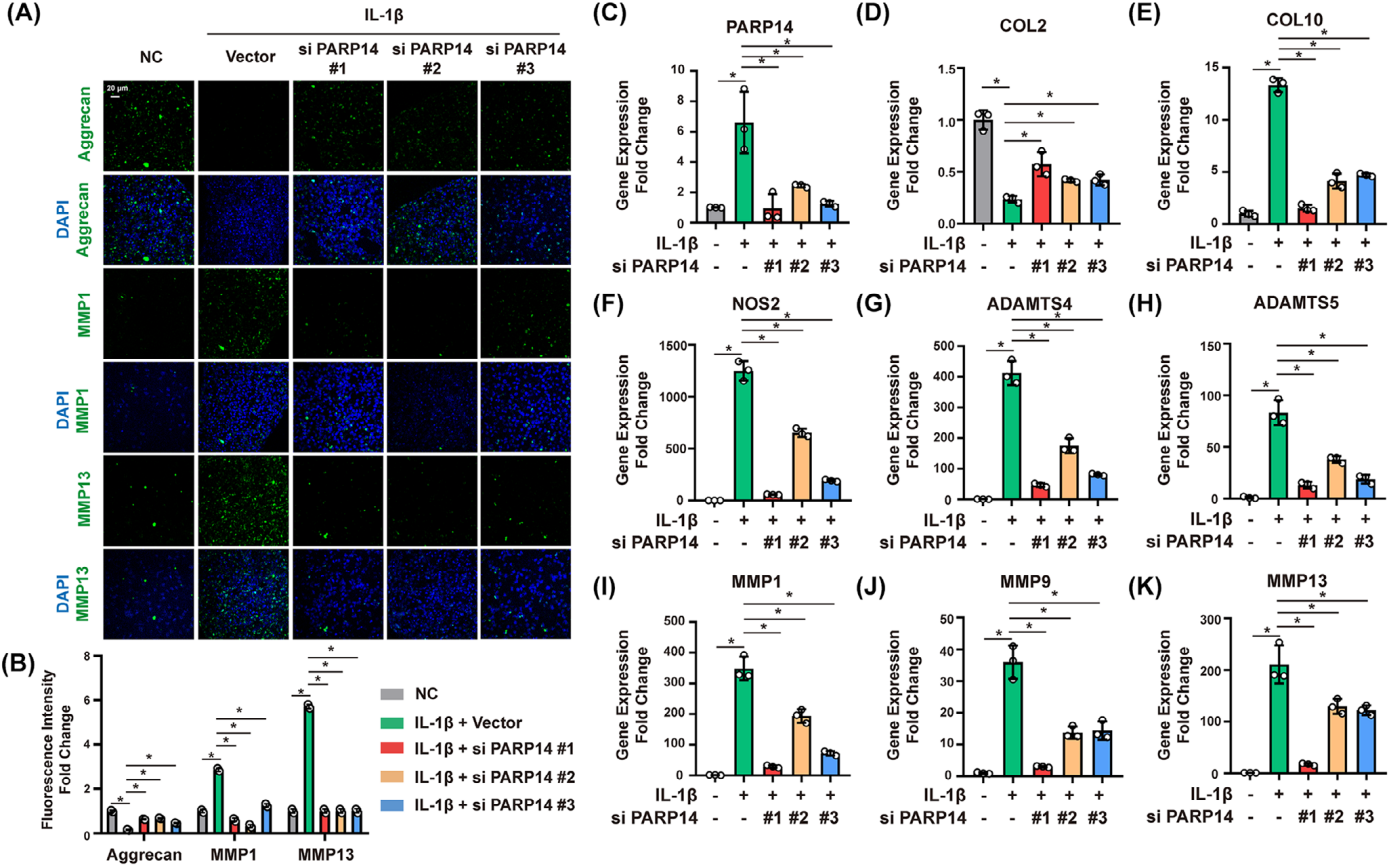
PARP14 regulates the metabolism of chondrocytes by regulating catabolic and anabolic markers. (A) Immunofluorescence staining for Aggrecan, MMP1, and MMP13 in primary chondrocytes transfected with PARP14 siRNA #1/#2/#3 or a negative control siRNA (NC) in the presence of IL‐1β. Scale bar = 20 µm. (B) Quantitative analysis of immunofluorescence. (C–K) PARP14, COL2, COL10, NOS2, ADAMTS4, ADAMTS5, MMP1, MMP9, and MMP13 mRNA levels in primary chondrocytes transfected with PARP14 siRNAs or an NC siRNA in the presence of IL‐1β. *n* = 3; *p < .05. All experiments were performed with at least three biological replicates and two technical replicates unless otherwise stated.

To test whether these impacts of PARP14 on cell metabolism were relevant to the pathophysiology of OA, we silenced PARP14 in primary chondrocytes by siRNA transfection to test for impacts on the extracellular matrix. PARP14 expression was silenced using siRNA transfection, and cells were subsequently exposed to IL‐1β to mimic an inflammatory environment. As expected, IL‐1β treatment alone markedly increased the expression of matrix‐degrading enzymes, including MMP1 and MMP13, while concomitantly reducing the expression of chondrogenic genes such as aggrecan (Figure 5A and B). When PARP14 was silenced, RT‐qPCR analysis confirmed a robust reduction in PARP14 mRNA expression and revealed a significant downregulation of multiple catabolic and hypertrophic markers, including Collagen X (COL10), A disintegrin and metalloproteinase with thrombospondin motifs 4 (ADAMTS4), ADAMTS5, NOS2, MMP1, MMP9, MMP13 (Figure 5C–K). In contrast, anabolic markers such as collagen type II (COL2) were significantly upregulated (Figure 5D). These results demonstrate that suppression of PARP14 not only dampens matrix catabolism but also helps preserve chondrogenic gene expression, suggesting that PARP14 is a key regulator of the balance between catabolic and anabolic processes in cartilage.

**FIGURE 6:**
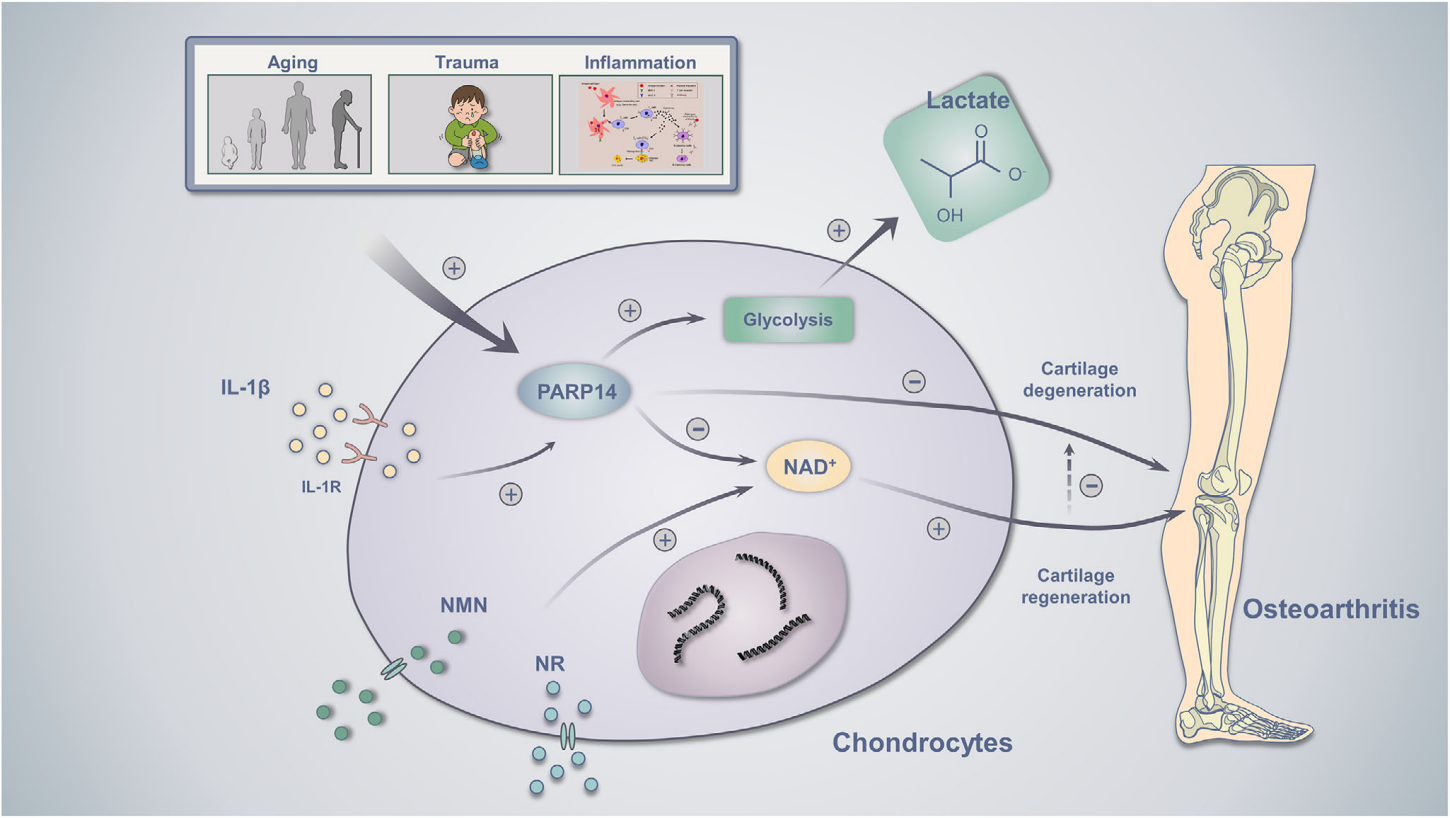
Graphical summary of our research.

Together, these results indicate that NAD^+^ and PARP14 are involved in OA pathogenesis, and their underlying mechanism is illustrated in Figure 6.

## 3 DISCUSSION

OA progression is characterised by metabolic imbalance within the joint, wherein catabolic activity surpasses anabolic processes, leading to extracellular matrix degradation and cartilage degeneration.^19^ While prior studies have suggested a decline in NAD⁺ levels with aging and inflammation,^20^, ^21^, ^22^ its direct role in OA pathogenesis remained unclear. Here, we demonstrate that impaired NAD⁺ homeostasis in OA is associated with increased consumption, particularly through PARP14, together with potential limitations in biosynthesis at the NMNATs step. Our findings identify PARP14 as a major NAD⁺‐consuming enzyme upregulated in OA cartilage and show that both pharmacological and genetic interventions of NAD⁺ attenuate cartilage damage in models of aging and post‐traumatic OA. Targeting PARP14 and NAD⁺ metabolism offers a potential disease‐modifying strategy for OA, addressing a major unmet clinical need.

Notably, NAMPT also circulates as an adipokine (eNAMPT/visfatin), linking systemic inflammation and metabolism to joint degeneration, and may complement our tissue findings.^23^ The paradox of elevated NAMPT but reduced NAD⁺ highlights the potential involvement of downstream NMNAT enzymes, whose impaired expression could compromise cytoplasmic and mitochondrial NAD⁺ synthesis. In parallel, NAD⁺ depletion in OA may be aggravated by excessive PARP activity, and studies suggest PARP inhibitors could preserve NAD⁺ and attenuate cartilage degeneration.^7^ Beyond NAD⁺ consumption, PARP14 also modulates macrophage polarisation and inflammatory signalling, underscoring its broader role in OA pathogenesis.^24^ Supporting this, NMNAT1 overexpression restored NAD⁺ levels and significantly protected against age‐related cartilage degeneration. These findings pinpoint NMNAT1‐mediated conversion of NMN to NAD⁺ as a critical regulatory step in maintaining NAD⁺ homeostasis during OA. The accumulation of NAM and NMN, alongside NAD⁺ decline, suggests a possible bottleneck at the NMNAT step; however, direct enzymatic activity measurements are needed to confirm this mechanism. This aligns with recent studies highlighting the importance of NAD⁺ biosynthesis in tissue health. Interestingly, Song et al.^25^ reported that liver‐specific knockout of NMNAT1 in mice did not significantly alter total hepatic NAD⁺ levels or compromise liver function. This finding implies the presence of compensatory mechanisms or alternative NAD⁺ biosynthetic pathways in hepatic tissue, underscoring the tissue‐specific roles of NMNAT1. In contrast, our study reveals that in cartilage tissue, overexpression of NMNAT1 is capable to protect cartilage degeneration against aging, underscoring the potential of targeting NAD⁺ biosynthetic pathways for therapeutic interventions. Meanwhile, future targeted metabolomics including reduced precursors (e.g., NR/NRH and NMNH) will be valuable to further dissect the contribution of different salvage routes to NAD⁺ homeostasis in OA.

Our integrated analyses consistently identified PARP14 as the dominant NAD⁺‑consuming enzyme in osteoarthritic cartilage. RNA‑seq of human G1 versus G4 cartilage revealed a significant upregulation of PARP14 compared with other NAD⁺‑consuming enzymes, including PARPs, CD38 and SIRTs. This transcriptional increase was corroborated by RT‑qPCR validation and immunohistochemical staining, which demonstrated higher PARP14 expression in chondrocytes from human OA cartilage, aged murine joints, and the rat MCL‑MM surgical OA model. Silencing PARP14 reduced glycolytic flux, and suppressed the expression of matrix‐degrading enzymes in IL‐1β‐stimulated chondrocytes. These results position PARP14 as a key driver of the metabolic reprogramming associated with OA pathology. While previous studies have reported similar effects for CD38 in OA, our data uniquely implicate PARP14 as a dominant NAD⁺‐consuming enzyme in this context.^6^ Notably, PARP14 inhibition suppressed the Warburg‐like glycolysis shift in inflamed chondrocytes, aligning with its role in cancer metabolism. However, parameters such as coupling efficiency, spare respiratory capacity, mitochondrial membrane potential, and reactive oxygen species (ROS) were not fully explored. Further in‐depth analyses are needed to delineate mitochondrial function and bioenergetic health. In addition, the effects of direct NAD⁺ precursor treatment on these metabolic parameters were not analysed in parallel and will be a focus of future investigations. While NAD⁺ depletion in skeletal muscle has been reported not to impair tissue integrity, our findings reveal that cartilage is uniquely vulnerable to NAD⁺ dysregulation, underscoring tissue‐specific roles of NAD⁺ metabolism.^26^ Although our data identify PARP14 as a key NAD⁺‑consuming enzyme in OA cartilage, the causal role of PARP14 in vivo has not yet been fully established. Future studies employing cartilage‑specific or inducible PARP14 knockout models will be essential to confirm its pathogenic contribution and to exclude potential off‑target effects observed in siRNA‑based assays.

Importantly, our data establish that restoring NAD⁺ levels via NMN or NR supplementation leads to significant chondroprotection, including reduced cartilage matrix loss, upregulation of chondrogenic markers (e.g., COL2), and downregulation of catabolic genes (e.g., MMP13, ADAMTS5). These effects were consistent across bovine explants, surgically induced OA rats, and naturally aged mice, suggesting broad therapeutic applicability. Moreover, the observation that NMNAT1‐transgenic mice exhibited both higher NAD⁺ levels and resistance to age‐related OA further supports the centrality of NAD⁺ metabolism in cartilage homeostasis. This is in line with findings from Song et al., who reported that nicotinic acid riboside supplementation not only restores tissue NAD⁺ levels but also improves metabolic dysfunction and aging‐related conditions, highlighting the therapeutic potential of NAD⁺ precursors in age‐associated diseases.^25^ Furthermore, our findings resonate with recent research demonstrating that ergothioneine, a naturally occurring antioxidant, significantly enhances NAD⁺ levels and extends lifespan in model organisms. Specifically, a study reported that ergothioneine supplementation in *Caenorhabditis elegans* led to a 20% increase in lifespan, attributed to enhanced NAD⁺ biosynthesis and improved mitochondrial function.^27^ This underscores the broader relevance of NAD⁺ augmentation strategies in promoting tissue regeneration and combating age‐related degeneration across species. Oral NAD⁺ precursors are clinically feasible, safe, and already under investigation in other aging‐related diseases, supporting future clinical translation for OA therapy.

While our study reveals a potential link between NAD⁺ metabolism and OA pathophysiology, future studies are needed to assess the long‐term safety and efficacy of NAD⁺‐boosting strategies in clinical settings. In particular, in vivo studies using conditional PARP14 knockout models could provide deeper mechanistic insights while avoiding potential embryonic lethality. Additionally, exploring the interplay between NAD⁺ metabolism and other cellular pathways involved in OA could uncover synergistic targets for intervention. Thirdly, we acknowledge that functional outcome measures such as pain‐related behaviour, gait analysis, and joint mobility were not assessed in this study. These readouts are essential to establish the translational relevance of NAD⁺‐modulating interventions. Future studies incorporating standardised behavioural assessments in preclinical OA models will be critical to validate whether structural improvements translate into meaningful functional benefit.

In conclusion, we identify disrupted NAD⁺ metabolism, driven by enhanced PARP14 expression and insufficient NMNAT1 function, as a potential, targetable mechanism underlying OA progression. Our findings suggest that NAD⁺ precursors and NMNAT1 modulation can preserve cartilage integrity at the histological and molecular levels in preclinical models, highlighting NAD⁺ metabolism as a potential target for future translational research, while definitive therapeutic efficacy requires further validation. These insights contribute to a growing body of evidence emphasising the critical role of NAD⁺ homeostasis in maintaining tissue health and combating degenerative diseases.

## 4 METHODS

### 4.1 Classification of tissues into OA and non‐OA

Clinical femur cartilage specimens were obtained from patients undergoing primary total knee arthroplasty. Human ethics approval for this project was granted by the Queensland University of Technology (QUT) and the St Vincent Private Hospital Ethics Committees, and informed consent was obtained from all participants (ethics number: #1400001024). Following the Kellgren and Lawrence grading system, the cartilage tissues collected from the relatively smooth region were classified into the grade 1 (G1, non‐OA) group, and the cartilage collected from the severely damaged region were classified into the grade 4 cartilage (G4, OA) group (*n* = 19; age: 72.5 ± 5.72 years, gender: 9 males and 10 females; BMI: 29.05 ± 5.31 kg/m^2^). Both G1 and G4 cartilages were harvested from each participant.

### 4.2 Primary culture of articular chondrocytes

For cell culture, cartilage was cut into small pieces and washed 3–4 times with phosphate‐buffered saline (PBS; Oxoid, Thermo Fisher Scientific, Victoria, Australia, CAT. OXBR0014G) to remove the blood debris. Chondrocytes were isolated by digestion with 1% type II collagenase (Gibco, Thermo Fisher Scientific, CAT. 17101015) for 6–10 h and resuspended in low glucose Dulbecco's Modified Eagle's Medium (DMEM; Gibco, CAT. 11885084) with 10% Fetal Bovine Serum (FBS; Gibco, CAT. A5256701) and 1% penicillin–streptomycin (Gibco, CAT. 15140122) (complete medium) as described previously.^28^ For 3‐dimensional (3D) pellet culture, 5 × 10^5^ of primary chondrocytes were centrifuged to form a 3D pellet as described previously.^28^ Pellets were cultured in chondrogenic culture medium (DMEM), high (4.5 g/L) glucose (DMEM; Gibco, CAT. 11965092) supplemented with 1% inulin–transferrin–selenium (Gibco, CAT. 41400045), 1.25 mg/mL bovine serum albumin (Sigma‐Aldrich, CAT. A3311),.1 µM dexamethasone (Sigma‐Aldrich, CAT. D4902),.1 mM ascorbic acid (Sigma‐Aldrich, CAT. A92902), 1% penicillin–streptomycin, 10 mM hydroxyethyl piperazineethanesulphonic acid (HEPES) (Gibco, CAT. 11560496),.1 mM L‐proline (Sigma‐Aldrich, CAT. P0380),.1 mM MEM nonessential amino acids (Gibco, CAT. 11140050), and 10 ng/mL transforming growth factor‐β1 (TGF‐β1, Gibco, CAT. 10021100UG) for 14 days according to our previous study.^28^

### 4.3 Experimental OA animal models

Ageing mice model was performed using young (6 weeks), middle‐aged (13 months), and aged (25 months) male C57/BL6 mice. Transgenic mice models were performed using wild type (WT, 13 months) and NMNAT1 transgenic C57/BL6 mice (NMNAT1 tg, 13 months). Mice were obtained from Dr Lindsay Wu's Laboratories (ethic number: 18/133A). For nicotinamide mononucleotide (NMN) treatment, 13‐month‐old mice were given both.5 and 2 g/L of NMN in drinking water for 4 weeks. For OA surgery model, 8‐week‐old male Wistar rats were purchased from the Animal Resources Centre (Perth, WA, Australia) and divided into four groups: Sham, Sham + nicotinamide riboside (NR, Tru Niagen, CAT. 856029008554), medial collateral ligament‐medial meniscus (MCL‐MM), and MCL‐MM + NR (ethic number: 1900000938, approved by University Animal Ethics Committee, QUT). Right knee of each rat was operated sham or MCL‐MM surgery.^29^ After that, Sham + NR and MCL‐MM + NR groups were given 200 mg/kg NR oral drop supplement daily for 8 weeks. Mice and rats were group‐housed (6 mice or 3 rats/cage) in ventilated double‐decker cages in a temperature‐controlled room on 12‐h light/dark cycles with ad libitum access to food and water and routine veterinary assessment. Animals were randomly assigned to experimental groups using a random number generator to minimise bias.

### 4.4 RNA sequencing (RNA‐Seq) library preparation and data analysis

Total RNA from G1/G4 cartilage (*n* = 10) was isolated and tested for integrity with Agilent's Fragment Analyser 5200 (Agilent Technologies, California, United States). RNA‐Seq libraries were then constructed using Qubit 4.0 Fluorometer (Thermo Fisher Scientific) with Qubit dsDNA High Sensitivity Assay Kit (Thermo Fisher Scientific, CAT. Q32851) and checked for quality with the Fragment Analyzer (Agilent Technologies). These were then pooled based on equimolar concentrations and sequenced on the Novaseq 6000 Sequencing System (Illumina) with an S1 PE100 sequencing kit (Illumina). High‐quality reads were then mapped onto the Human Genome Reference Consortium build 38 using Spliced Transcripts Alignment to a Reference (STAR, version 2.7.9a) and feature counts extracted for human Ensembl annotated genes (release 103). Differential gene expression (DE) analysis was conducted using DESeq2 (Bioconductor, version 1.32.0) with an FDR < .05.

### 4.5 Histological analysis and immunohistochemistry (IHC)

Staining was performed following the methods from our previous publication.^30^ All histological scoring and outcome assessments were performed by investigators blinded to group allocation. Both cartilage and knee joints were fixed in 4% paraformaldehyde (PFA; Sigma‐Aldrich, CAT. P6148), decalcified in 10% ethylenediaminetetraacetic acid (EDTA; Sigma‐Aldrich, CAT. E5134) and embedded in paraffin. All paraffin samples were cut into 5 µm sections. For Safranin O/Fast Green staining, samples were stained with.1% Safranin O solution (Sigma‐Aldrich, CAT. TMS‐009‐C) and 1% Fast Green solution (Sigma‐Aldrich, CAT. F7252). Modified mankin score was used to grade the severity of cartilage degeneration by two observers blinded to group‐identifying information. For Masson's Trichrome staining, samples were stained with Masson's composition solution (Electron Microscopy Sciences, CAT. 26367) and aniline blue solution (Electron Microscopy Sciences, CAT. 50‐317‐77). The percentage of collagen area was calculated using Image J imaging software (version 1.51n). For IHC staining, samples were stained with primary monoclonal antibodies and secondary antibody (EnVision Dual Link, Dako, CAT. K4065). Positively stained cells were evaluated using Image J. The primary antibodies used were as follows: anti‐NAMPT antibody (1:200, Abcam, Cambridge, United Kingdom, ab45890), anti‐DIPEN antibody (1:500, kind gift from Professor Amanda Fosang, Murdoch Childrens Research Institute, Melbourne, VIC, Australia), anti‐NITEGE antibody (1:500, kind gift from Professor Amanda Fosang, Murdoch Childrens Research Institute, Melbourne, VIC, Australia), anti‐COL2 antibody (1:200, Abcam, ab34712), anti‐MMP13 antibody (1:200, Abcam, ab39012), anti‐PARP14 antibody (1:200, Santa Cruz Biotechnology, Texas, United States, sc‐377150).

### 4.6 Nicotinamide adenine dinucleotide (NAD^+^)/NAD^+^ hydrogen (NADH) assay

Cartilage sample was grounded into powder in liquid nitrogen and homogenised with NAD/NADH Extraction Buffer. The supernatant was collected and filtered through a 10 kD spin column (Abcam, CAT. ab93349). NAD^+^ and total NAD (tNAD) levels were measured using NAD/NADH Assay Kit (Colorimetric; Abcam, CAT. ab65348) according to the manufacturer's instructions. Reading was taken at Optical Density (OD) 450 nm.

### 4.7 Real‐time quantitative polymerase chain reaction (RT‐qPCR)

Total RNA was extracted using TRIzol reagent following our previous publication.^30^ Reverse transcription of mRNAs to cDNA was performed using miScript PCR Starter Kit (Qiagen, Hilden, Germany, CAT. 339320). RT‐qPCR was used to determine the mRNA expression levels using the gene‐specific primers (Sigma‐Aldrich) with SYBR Green (Life Technologies, California, United States, CAT. A25742) on QuantStudio Real‐Time PCR system (Applied Biosystems, Massachusetts, United States). The relative mRNA expression levels were quantified using the 2^−ΔΔCt^ method. All RT‐qPCR experiments were performed in triplicate. All primers are listed in Table S1.

### 4.8 Western blot

Western blot was performed following our previous study.^28^ SDS‐PAGE separated total cellular protein, then transferred to a Nitrocellulose Transfer membrane (.2 µm pore size, BioTrace, Arizona, United States, CAT. 27376–991). The membranes were blocked and then incubated with primary antibodies overnight at 4°C, and subsequently incubated with secondary antibodies for 1 h. α‐tubulin served as the internal standard, and Image J was used to quantify blots intensities. The antibodies used were as follows: anti‐NAMPT antibody (1:1000), anti‐KNYU antibody (1:1000, Abcam, ab225916), anti‐IDO1 antibody (1:1000, Abcam, ab55305), anti‐QPRT antibody (1:1000, Abcam, ab171944), anti‐3‐HAAO antibody (1:1000, Abcam, ab106436), anti‐NMNAT1 antibody (1:1000, Abcam, ab118270), anti‐α‐tubulin antibody (1:1000, Abcam, ab7291).

### 4.9 Metabolite extraction and LC‐MS/MS of intracellular metabolites

50 mg of each cartilage sample was grounded into powder in liquid nitrogen and homogenised using Omni TH Homogenizer (Omni, Inc, Georgia, United States). The supernatant was collected, and 1 mL of chloroform (Sigma‐Aldrich, CAT. C2432) was added, incubated on ice and centrifuged for 10 min at 13 000 × *g* at 4°C. The supernatant was then collected, freeze‐dried, and resuspended in 90 µL of 1% ACN in water for LC‐MS/MS analysis.

Kynurenine pathway metabolites were quantified by LC‐MS/MS. In brief, a targeted LC‐MS/MS metabolomics analysis for kynurenine pathway metabolites was performed using a Shimadzu UHPLC System coupled to a Shimadzu 8060 triple quadrupole (QqQ) mass spectrometer. The UHPLC (Nexera X2, Shimadzu Corp., Kyoto, Japan) consisted of LC‐30AD pump units, DGU‐20ASR degassing units, a SIL‐30AC autosampler, a CTO‐20AC column oven, a CBM‐20A communications BUS module and an FCV‐20AH2 diverter valve unit. LC was performed using a Phenomenex Gemini‐NX C18 column (150 × 2 mm, 3 µm, 110 A, PN: 00F‐4453‐B0) with a guard column (Phenomenex SecurityGuard Gemini‐NX C18, 4 × 2 mm, PN: AJ0‐8367). Solvents A and B were.1% v/v formic acid (Optima LC/MS Grade, Fisher Chemicals) in high purity water (Milli‐Q Reference Water Purification System, Merck) and in acetonitrile (Lichrosolv, PN: 1142914000, Merck), respectively. A flow rate of 300 µL/min was used to run the HPLC gradient, which was as follows: 2% B from 0–1 min, 2%–20% B from 1–22 min, 20%–98% B from 22–28 min, 98% B continued to 34 min, then 2% B at 35–40 min. Samples were kept at 4°C in the autosampler, and the column was operated at 50°C in a column oven.

The Shimadzu 8060 QqQ system had an electrospray ion source (ESI), which used nitrogen (> 99.999 vol % BOC, NSW, Australia) as drying gas, and argon (> 99.999 vol %, UN1006, Coregas Pty Ltd, NSW, Australia) as collision gas. Further instrument detail includes drying gas flow: 10 L/min, nebulising gas flow: 3.0 L/min, heating gas flow: 10 L/min, desolvation line: 250°C, heat block temperature: 400°C, CID gas: 270 kPa, and interface temperature: 300°C. Data were collected from 1–20 min, while the flow eluent was diverted to waste at 0–1 min and 22–40 min during column clean up and re‐equilibration.

Multiple reaction monitoring (MRM) transitions were optimised on either the negative or positive ionisation mode (*m*/*z*‐H or *m*/*z*+H). All analytical standards were purchased from Sigma‐Aldrich and stock solutions were prepared using high purity water. Details of the compound‐ and instrument‐specific parameters are shown in Table S2.

### 4.10 Cartilage explant harvest

Cartilage explants were obtained from the femoral condyle head of fresh young bovine knee joints (3–5 months old) using a 3.0 mm biopsy punch (Kai Medical, Gifu, Japan, CAT. BP‐30F).^31^ After that, explants were cultured in a chondrogenic culture medium at 37°C for 72 h before further experiments. For NMN treatment, cartilage explants were washed with 1× PBS 3–4 times, and the culture medium was changed to chondrogenic culture medium with/without 1 mM NMN and 10 ng/mL IL‐1β for 48 h.

### 4.11 1,9‐Dimethylmethylene blue (DMMB) assay

Glycosaminoglycan (GAG) was measured following the protocol of DMMB assay with purified shark chondroitin sulphate (Sigma‐Aldrich, CAT. C6737) as a standard.^32^ In brief, for GAG release, 30 µL of cartilage explants culture media was added to 96‐well plates with 300 µL of DMMB dye solution (Sigma‐Aldrich, CAT. 341088). All samples and standards were read at a 525 nm wavelength. Cartilage explants were dried and weighed. The percentage of GAG release was calculated as GAG release per tissue weight.

### 4.12 siRNA transfection

The PARP14 siRNAs and negative control vector were transfected into primary chondrocytes, C28/I2 chondrocytes cell line and 3D pellets for 24–72 h using the Lipofectamine RNAiMAX (ThermoFisher Scientific, CAT. 11668027) according to the recommended protocol by the manufacturer. The target sequences of siRNAs were as follows: siPARP14 (#1), 5′‐GGCTGAATGGTGCTTGGTACA‐3′; siPARP14 (#2), 5′‐ GCTGAATGGTGCTTGGTACAA‐3′; siPARP14 (#3), 5′‐ GCCTCATTTCATCGTTTATCT‐3′.

### 4.13 Cell counting kit 8 (CCK‐8) assay

Chondrocytes were seeded at a density of 5 × 10^3^ cells/well in 96‐well plates. The next day, cells were transfected with siPARP14 or negative control vector for 24, 48, and 72 h. CCK‐8 assay (Abcam, CAT. ab228554) was performed according to the manufacturer's instructions. After each time point, 10 µL of WST‐8 solution was added to each well and incubated for 1 h at 37°C in the dark. Absorbance was measured at 460 nm.

### 4.14 Measurement of extracellular metabolites

2 × 10^6^ of primary P0, G1, and G4 chondrocytes were seeded on each T75 culture flask with a complete medium. On the next day, cells were transfected using siPARP14 or negative control vector with/without 10 ng/mL IL‐1β for 48 h. 1 mL of culture medium was collected in each flask after that. The quantification of glucose and lactate was performed using a previously published method with minor modifications. Glucose, pyruvate, and lactate were quantified by ion‐exclusion chromatography using an Agilent 1200 high‐performance liquid chromatography (HPLC) system with UV and RI detectors (Agilent Technologies) and an Agilent Hi‐Plex H column (300 × 7.7 mm; Agilent Technologies) with a guard column (SecurityGuard Carbo‐H, 4 × 3 mm, Phenomenex, New South Wales, Australia).^33^ Chromatograms were integrated using the Chromeleon software (version 7.3, Thermo Scientific Dionex).

### 4.15 Measurement of cellular bioenergetics by Seahorse assays

Chondrocytes were seeded at a density of 5 × 10^3^ cells/well in Seahorse XF96 Cell Culture Microplates (Agilent Technologies, CAT. 103794‐100). The next day, cells were transfected with siPARP14 or negative control vector with/without 10 ng/mL IL‐1β for 48 h. After that, the Glycolysis Stress test (Agilent Technologies, CAT. 103020–100) and Mito Stress test (Agilent Technologies, CAT. 103015–100) were performed according to the manufacturer's instructions.^28^ For the Glycolysis Stress test, cells were sequentially treated with 10 mM glucose, 1 µM oligomycin, and 50 mM 2‐deoxyglucose (2DG). For the Mito Stress test, cells were sequentially treated with 1.5 µM oligomycin, 1 µM carbonyl cyanide‐4 (trifluoromethoxy) phenylhydrazone (FCCP), and a combination of.5 µM rotenone/antimycin A. The following methodology was used to collect each set of measurements: mix for 3 min, measure for 3 min, repeat cycle 3–4 times. All cells were plated in triplicates. Each assay was performed twice. Prior to analysis, zeros were removed following Agilent's guidelines. We averaged the remaining replicates. Calculations were made using the average of all measurements per injection and normalised to total protein content using BCA protein assay (Pierce, Thermo Fisher Scientific, CAT. 23227). Data were analysed using Wave Desktop 2.6 (Agilent Technologies).

### 4.16 Immunofluorescence (IF) staining

Chondrocyte pellets and C28/I2 cells were fixed in 4% PFA for 30 min. After that, pellets were embedded in paraffin, sectioned into 5 µm of slides and dewaxed. Then, sections were incubated with proteinase K and 3% H_2_O_2_ for 20 min, respectively. C28/I2 cells were covered with 1× PBS containing.25% Triton X‐100 for 15 min. Next, both pellets sections and cells were sealed with 1% BSA for 1 h. After that, sections and cells were incubated with primary antibodies overnight at 4°C and secondary antibodies (Goat Anti‐Rabbit, Alexa Fluor 488, Abcam, ab150077) for 1 h at room temperature. Nuclei were restained with DAPI (Thermo Fisher Scientific, CAT. D3571) for 5 min. Images were captured using a confocal laser scanning microscope (Nikon A1R Confocal, Amsterdam, The Netherlands). Fluorescence intensity was measured using Image J. The antibodies used were as follows: anti‐Aggrecan antibody (1:200, Abcam, ab186414), anti‐MMP1 antibody (1:200, Abcam, ab52631), anti‐MMP13 antibody (1:200).

### 4.17 Statistical analysis

All experiments were performed in triplicate using different human samples collected from 5 different donors (5 independent experiments) except 6 donors for NAD^+^/tNAD measurement and RT‐qPCR, 10 donors for RNA sequencing, and 3 donors for siRNAs‐related experiments. All animal experiments were performed using 6 animals per group except 3 samples per group for bovine explants experiments. The statistical analysis was performed using GraphPad Prism (version 8, California, United States), graphically testing for normal distribution, and using either *t*‐test or one‐way ANOVA with Tukey's post hoc analysis. The data are presented as mean ± standard deviation (SD) for all variables. For all figures, differences were considered significant with *p* < .05.

## AUTHOR CONTRIBUTIONS

XW and IP conceived and designed the project. XW, XF, MJB, DMG, and LEW performed all cell and animal experiments. XW, MP, TS, TM, and RB collected and analysed the data. XW drafted the manuscript. XM draw the graphic summary. RC and IP supervised the project. All authors revised the manuscript.

## CONFLICT OF INTEREST STATEMENT

LEW is a co‐founder, shareholder and consultant to Jumpstart Fertility, which is developing NAD boosting molecules to enhance assisted reproductive technology, and in Metro Biotech, which is developing NAD boosters for the treatment of age‐related disease. Experiments in Figure 3H–M performed in the lab of LEW were in part supported by sponsored research funding from Jumpstart Fertility Pty Ltd.

## PATIENT CONSENT FOR PUBLICATION

Obtained.

## ETHICS STATEMENT

Queensland University of Technology (QUT), the St Vincent Private Hospital Ethics Committees, University Animal Ethics Committee in QUT and the University of New South Wales approved this study (Ethics number: #1400001024, 1900000938, 18/133A).

## Supporting information



Supporting Information

## Data Availability

All data generated or analysed during this study are included in this article and its supplementary information files. All other data collected and analysed during the current study are available from the corresponding author upon reasonable request.
