# Supplementary material for: Altered nicotinamide adenine dinucleotide metabolism drives cartilage degeneration and osteoarthritis
Source: Clin Transl Med. 2025 Nov 6;15(11):e70513. doi: 10.1002/ctm2.70513 (PMC12589899; doi:10.1002/ctm2.70513)
Supplement: Supplementary file 1 — Supporting Information [file CTM2-15-e70513-s001.docx]

Supplementary Materials for

**Altered Nicotinamide Adenine Dinucleotide Metabolism Drives Cartilage Degeneration and Osteoarthritis**

**Xiaoxin Wu *et al.***

**
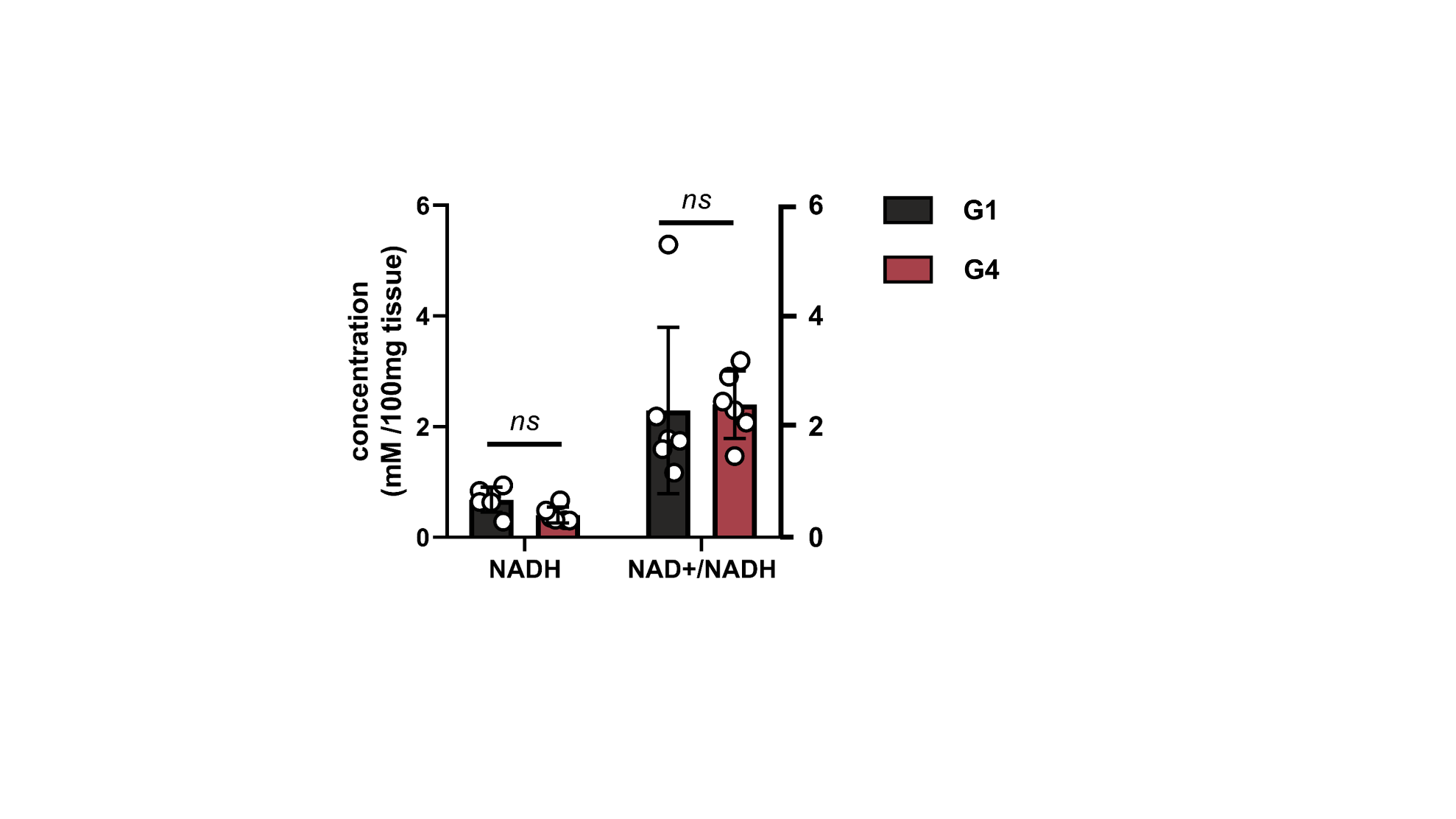
**

**Supplementary fig. S1**

Levels of NADH and NAD⁺/NADH ratio in G1 and G4 cartilage. Ns, non-significant; n = 6.

**
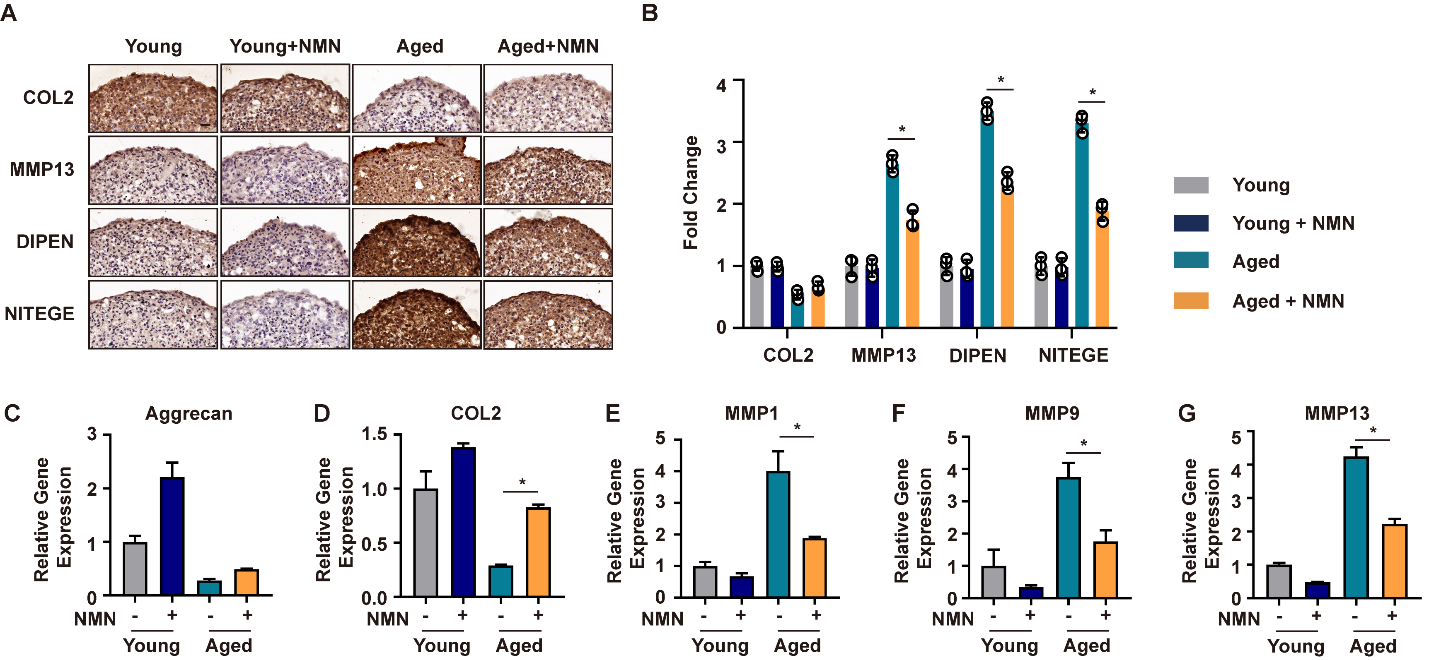
**

**Supplementary fig. S2**

**(A)** Immunofluorescence analysis of COL2, MMP13, DIPEN, and NITEGE expression on 3D cultured primary chondrocytes from both young and old donors under NMN treatment. **(B)** Immunofluorescence analysis quantification. **p* ≤ 0.05; n = 3. **(C)** Aggrecan, COL2, MMP1, MMP9, and MMP13 mRNA levels in primary chondrocytes treated with NMN. **p* ≤ 0.05; n = 3.


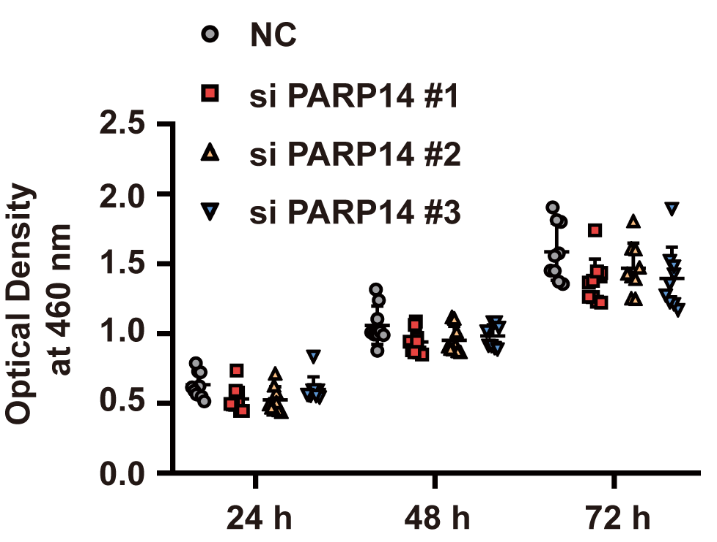


**Supplementary fig. S3**

Cell counting kit-8 (CCK-8) assay of silencing PARP14 expression on chondrocytes for 24, 48, and 72 hours. n = 9.

**
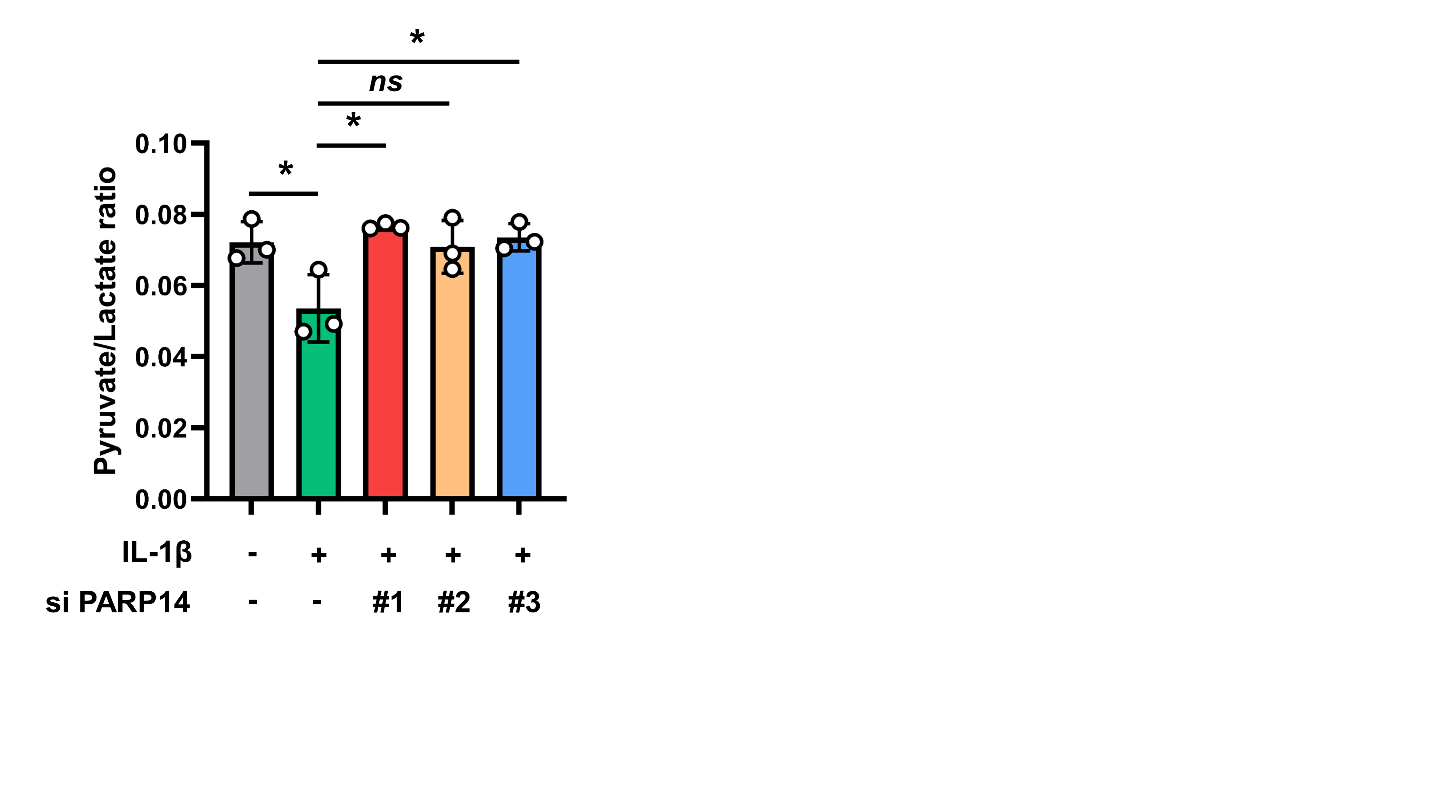
**

**Supplementary fig. S4**

Pyruvate/lactate ratio in chondrocytes transfected with PARP14 siRNAs or NC siRNAs. n = 3.

**Supplementary table S1. Sequences of gene-specific primers used in the study**

| **Primers** | **Sequences (5’ → 3’)** |
| --- | --- |
| MMP1 F | AAAATTACACGCCAGATTTGCC |
| MMP1 R | GGTGTGACATTACTCCAGAGTTG |
| MMP9 F | GGGACGCAGACATCGTCATC |
| MMP9 R | TCGTCATCGTCGAAATGGGC |
| MMP13 F | TCCTGATGTGGGTGAATACAATG |
| MMP13 R | GCCATCGTGAAGTCTGGTAAAAT |
| COL2 F | TGGACGATCAGGCGAAACC |
| COL2 R | GCTGCGGATGCTCTCAATCT |
| COL10 F | ATGCTGCCACAAATACCCTTT |
| COL10 R | GGTAGTGGGCCTTTTATGCCT |
| NOS2 F | TTCAGTATCACAACCTCAGCAAG |
| NOS2 R | TGGACCTGCAAGTTAAAATCCC |
| ADAMTS4 F | GAGGAGGAGATCGTGTTTCCA |
| ADAMTS4 R | CCAGCTCTAGTAGCAGCGTC |
| ADAMTS5 F | GAACATCGACCAACTCTACTCCG |
| ADAMTS5 R | CAATGCCCACCGAACCATCT |
| AGGRECAN F | ACTCTGGGTTTTCGTGACTCT |
| AGGRECAN R | ACACTCAGCGAGTTGTCATGG |
| PARP14 F | TGTTAGTGGAGAACATAAGTGGC |
| PARP14 R | TGAATGGTGCTTGGTACAATCAT |
| KNYU F | GGCTCTCCACCTAGATGAGGA |
| KNYU R | GCTGCTATTTTGGCCCACTTAT |
| 3-HAAO F | AGGACTATCACATCGAAGAGGG |
| 3-HAAO R | ATGACCACATCCCGGTGTTTC |
| NADS F | AGGAGCAAGATACAGGCTTGG |
| NADS R | GTCCGACTCGTAATAATGATCCC |
| QPRT F | GGGCAGCCTTTCTTCGATG |
| QPRT R | GGAGCCCATACTTCTCCACCA |
| NAMPT F | CGGCAGAAGCCGAGTTCAA |
| NAMPT R | GCTTGTGTTGGGTGGATATTGTT |
| NMNAT1 F | TCTCCTTGCTTGTGGTTCATTC |
| NMNAT1 R | TGACAACTGTGTACCTTCCTGTT |
| GAPDH F | GGAGCGAGATCCCTCCAAAAT |
| GAPDH R | GGCTGTTGTCATACTTCTCATGG |

**Supplementary table S2.** **Metabolite-specific parameters used in the acquisition of the sMRM data**

| **Metabolite** | **Q1**  **(m/z)** | **Q3**  **(m/z)** | **RT**  **(min)** | **Dwell time (msec)** | **Q1 pre bias**  **(V)** | **Collision energy**  **(V)** | **Q3 pre bias**  **(V)** |
| --- | --- | --- | --- | --- | --- | --- | --- |
| NAD | 663.90 | 136.00 | 2.0 | 25 | -30 | -50.2 | -20 |
|  | 663.90 | 428.05 | 2.0 | 25 | -26 | -26.5 | -20 |
|  | 663.90 | 524.10 | 2.0 | 25 | -28 | -19.2 | -26 |
| TRP | 203.10 | 116.10 | 6.8 | 50 | 22 | 18.9 | 10 |
|  | 203.10 | 142.20 | 6.8 | 50 | 24 | 17.7 | 13 |
|  | 203.10 | 74.05 | 6.8 | 50 | 25 | 16.6 | 10 |
| NMN | 335.05 | 123.10 | 1.4 | 25 | -17 | -16.0 | -26 |
|  | 335.05 | 97.15 | 1.4 | 25 | -17 | -24.3 | -19 |
|  | 335.05 | 80.20 | 1.4 | 25 | -17 | -47.2 | -17 |
| NAM | 122.95 | 80.00 | 2.0 | 25 | -20 | -20.2 | -16 |
|  | 122.95 | 78.10 | 2.0 | 25 | -20 | -25.2 | -16 |
|  | 122.95 | 53.00 | 2.0 | 25 | -20 | -30.2 | -20 |
| KYN | 209.00 | 192.15 | 3.5 | 25 | -20 | -9.8 | -21 |
|  | 209.00 | 146.15 | 3.5 | 25 | -19 | -17.6 | -15 |
|  | 209.00 | 118.10 | 3.5 | 25 | -19 | -22.1 | -12 |
| NA | 124.15 | 78.10 | 1.9 | 25 | -19 | -21.8 | -15 |
|  | 124.15 | 80.15 | 1.9 | 25 | -19 | -23.1 | -15 |
|  | 124.15 | 53.10 | 1.9 | 25 | -13 | -28.6 | -21 |
| AZT | 266.20 | 223.15 | 16.8 | 50 | 12 | 10.3 | 22 |
|  | 266.20 | 42.05 | 16.8 | 50 | 12 | 23.3 | 14 |
|  | 266.20 | 84.05 | 16.8 | 50 | 12 | 11.3 | 11 |

***Note:*** Q: quadrupole, RT: retention time, V: volts
